# Study of Lightweight Ceramic Matrix-Less Syntactic Foam Composed of Cenosphere Using Spark Plasma Sintering

**DOI:** 10.3390/ma17020450

**Published:** 2024-01-17

**Authors:** Toms Valdemars Eiduks, Reinis Drunka, Vitalijs Abramovskis, Ilmars Zalite, Pavels Gavrilovs, Janis Baronins, Vjaceslavs Lapkovskis

**Affiliations:** 1 Institute of Materials and Surface Technologies, Riga Technical University, P. Valdena str. 7, LV-1048 Riga, Latvia; toms-valdemars.eiduks@rtu.lv (T.V.E.); reinis.drunka@rtu.lv (R.D.); ilmars.zalite@rtu.lv (I.Z.); 2 Laboratory of Ecological Solutions and Sustainable Development of Materials, Institute of General Chemical Engineering, Faculty of Materials Science and Applied Chemistry, Riga Technical University, Paula Valdena Street 3/7, LV-1048 Riga, Latvia; vitalijs.abramovskis@edu.rtu.lv (V.A.); pavels.gavrilovs@rtu.lv (P.G.); janis.baronins@rtu.lv (J.B.); 3 Institute of General Chemical Engineering, Faculty of Materials Science and Applied Chemistry, Riga Technical University, Paula Valdena Street 3/7, LV-1048 Riga, Latvia

**Keywords:** spark plasma sintering, matrixless syntactic foam, cenospheres, porosity, compressive strength, SiO_2_, XRD, shrinkage, apparent density

## Abstract

The current investigation presents porous ceramic materials prepared with cenospheres (CS) by using spark plasma sintering. The impact of sintering temperature, mould diameter (20, 30 and 50 mm) and cenosphere size on the properties of the sintered material was investigated. Shrinkage of the samples during sintering started at 900 °C. Total sample shrinkage during sintering increases with increasing temperature and decreases with increasing mould size; increasing sample sintering temperature increases the apparent density of all sample series CS 63–150 µm in a 20 mm mould from 0.97 to 2.3 g·cm^−3^ at 1050–1300 °C; in a 30 mm mould, 0.81–1.87 g·cm^−3^ at 1050–1200 °C; in 50 mm mould, 0.54–0.75 g·cm^−3^ at 1050–1150 °C; while CS 150–250 µm in a 20 mm mould is 0.93–1.96 g·cm^−3^ at 1050–1200 °C. Total porosity decreases from 61.5% to 3.9% by increasing sintering temperature from 1050 to 1250 °C, while open porosity reduces at lower temperatures, with closed porosity being highest in samples sintered at 1150 °C. When the sintering temperature increases from 1050 to 1300 °C, the compressive strength of the CS 63–150 samples produced in a 20 mm mould increases from 11 MPa to 312 MPa. These results correlate with the Rice model, which describes an exponential dependence of compressive strength on material porosity and fully dense material compressive strength.

## 1. Introduction

In the contemporary industrial landscape, there is a pressing need to innovate and produce lightweight and durable materials. This dual objective allows for creating more structurally efficient objects, necessitating reduced material usage, and enabling compact designs, and carries economic benefits. These benefits are realised through material savings and a reduction in the weight of objects. In the context of vehicles, this translates to energy conservation during their operation.

When developing a composite material, it becomes feasible to combine the distinctive properties of various materials into one [1]. To guarantee a high level of strength in the composite, it is essential to incorporate a material inherently to have high strength [2,3]. 

One of the promising material structures is a syntactic foam (SF). It is a type of composite material consisting of hollow microspheres embedded in a matrix material, typically a polymer of Al, Mg alloys [4,5,6].

It is known for its low density, high strength, and buoyancy, making it valuable in various industrial applications. The necessity of syntactic foam arises from its unique combination of properties, which offer several advantages in specific contexts. Here are some key reasons why syntactic foam is considered necessary in certain applications.

Buoyancy and Low Density. SF is lightweight and possesses excellent buoyancy due to the presence of hollow microspheres. This makes it ideal for applications where buoyancy is crucial, such as in underwater vehicles, buoys, and subsea equipment [7,8].

High Strength-to-Weight Ratio. Despite its low density, syntactic foam exhibits a high strength-to-weight ratio [9]. This makes it valuable in applications where both structural integrity and weight considerations are important, such as in marine structures, deep-sea exploration, and aerospace components.

Thermal Insulation. SF can provide thermal insulation in certain applications. This property is beneficial in industries like oil and gas, where equipment needs to maintain specific temperature conditions, or in cryogenic applications where insulation against extreme cold is required [7].

Reduced Water Absorption: SF typically has high porosity and pores are isolated–closed. This leads to low water absorption properties [8]. This makes it advantageous in marine and underwater applications where the material needs to maintain its buoyancy and structural integrity even when submerged for extended periods [10]. 

Customizable Properties: The composition of syntactic foam can be tailored to meet specific requirements. By adjusting the type and volume fraction of microspheres [9], as well as the matrix material, engineers can design syntactic foam with properties that suit the needs of a particular application [11].

However, the majority of SF is made of materials which cannot withstand the high temperatures (polymers [12,13], glass [14], Al and Mg [15]).

Concrete and ceramic matrices of SF, of course, are known and studied [16,17], but the influence of high temperatures on such materials also leads to its decomposition (concrete) and melting (glass softening temperature 550–650 °C). The necessity of new SF foam arises from its unique combination of properties, including buoyancy, high strength-to-weight ratio, thermal insulation, chemical resistance, and customizable characteristics. These features make syntactic foam a valuable material in industries such as marine engineering, aerospace, defence and underwater exploration. One of the promising components of SF are cenospheres (CS). A substantial quantity of ash, containing CS, is formed during coal combustion, which can then be processed using various methods to extract these particles. The separation of CS from the ash contributes to a reduction in waste produced by thermal power stations [18]. It offers an opportunity to harness these unique ceramic particles for composite material development. CS, derived from coal combustion residues, are available at a low cost as a component of fly ash from coal-fired power plants. They are characterised as chemically inert and resistant to high temperatures, making them a successful type of hollow ceramic microbead for use as filler particles. These CS have proven suitability, even in high-temperature processes [19]. The apparent density of CS ranges from 0.4 to 0.8 g·cm^−3^, although depending on the definition, i.e., whether CS are described as particles with an apparent density below that of water or as hollow particles, the maximum density will approach that of a solid ash particle, i.e., about 2.0–2.6 g·cm^−3^ [20]. This lower density is due to the gas bubble trapped in the centre of the particles, which has a composition similar to that of flue gases, and also the fact that for different particle sizes, the ratio of diameter to wall thickness is constant at around 20–30 [21]. The diameter of the CS ranges from 5 µm to over 600 µm, although most of the particles are between 20 µm and 300 µm in diameter [22].

Using the CS, it is possible to synthesise lightweight composites called syntactic foams (SF) with enhanced mechanical properties and reduced weight [23]. SF synthesis typically uses different matrix materials which are combined with hollow spherical spheres (or microspheres) like glass hollow-microbaloons [24], ceramic hollow spheres [25,26], or CS. In past decades CS has been actively studied for use in new SF designs using various matrices, polymer [27,28], ceramic [29,30], glass [31,32], metals [33,34], natural ceramics (clay) [35], cement [16,36], hybrid materials [37] and natural-sourced matrix [38]. However, a new version of SF, a matrix-less SF, was introduced. CS were coated with metal using physical vapour deposition and sintered by spark plasma sintering to obtain a single-piece material [39].

Spark Plasma Sintering (SPS) stands out for its ability to control microstructure and adapt heating parameters through pulsed direct current and pressure. In the case of conductive materials, the formation of inter-particle contacts includes local melting. When employed as a furnace, SPS facilitates high-rate heating for pre-consolidated specimens. Like other sintering techniques, SPS enables the creation of porous materials through methods such as partial densification, sintering hollow or porous particles, utilising decomposing pore formers, or employing space holders that are extracted post-sintering [40]. The SPS method has proven instrumental in producing advanced ceramics such as nanostructured ceramics, functionally graded materials (FGMs), ceramic matrix composites and nanocomposites. It proves to be an effective, non-conventional sintering method, ensuring the attainment of fully dense materials while preserving nanostructure features [41,42]. SPS can achieve homogenous, highly dense sintered materials at faster rates and lower temperatures, resulting in finer microstructures than conventional sintering methods [42]. In addition, SPS can be used to produce compact samples with high porosity (65–80%), which is higher than using traditional methods (porosity limit—50%) for analogical materials [23,43] and at the same time is possible to produce corrosion-resistant fibres [44] and demonstrate notably low thermal diffusivity, rendering them appealing for applications in thermal management [45]. SPS of porous materials is a rapidly advancing field, holding great promise for developing energy-absorption materials, bioimplants, high-temperature filters, fuel cells and thermoelectric materials [40].

In the current study, we explore the matrix-less SF made of CS compaction and sintering to determine sintering behaviour, distinguish the main sintering stages, dependences, and to characterise material properties are main goals of the work. For the first time, this work studied CS compaction behaviour at the SPS process. This study focuses on designing and characterising matrix-less SF containing only CS. The influence of sintering temperature, mould diameter (20, 30 and 50 mm), and the size of CS on the properties of sintered material was investigated. These primary data are essential for designing lightweight and thermally stable (up to 1000 °C) composite materials.

## 2. Materials and Methods

### 2.1. Raw Material Properties

The sintering material used in the study was the CS2 63–150 µm and CS1 150–250 µm fractions described in the paper [22], produced by burning coal from the Donetsk coal mine. The key data as chemical compositions of the C1 and CS2 shown in Table 1 and Table 2 represents data of bulk density, picnometric density and the interparticle void fraction of the CS1 and CS2. The resulting fly ash of CS1 and CS2 was flotated in a mixture of distilled water and ethanol, dried at 105 °C for 12 h, heat treated at 1100 °C for 30 min and fractionated into the above fractions via sieving. In CS1 and CS2, 46% and 44% of open volume fractions were observed in the burials of the prepared materials, respectively.

The samples were sintered in an SPS sintering machine “Dr. Sinter SPS 825” (SPS Syntex, Tokyo, Japan). The machine is designed for sintering materials at temperatures up to 2400 °C with a sintering force of up to 250 kN. However, in this study, CS were sintered from 1050 °C to 1300 °C with a minimally applied uniaxial pressing force, which differed between the used mould sizes, resulting in applied force being approximately 2.2, 1.4 and 0.9 MPa for the 20, 30 and 50 mm diameter moulds, respectively. These settings were chosen because of the relative brittleness of the material to be sintered, the CS, compared to homogeneous, dense, raw materials and the need to produce low-density ceramics.

**Figure 1:**
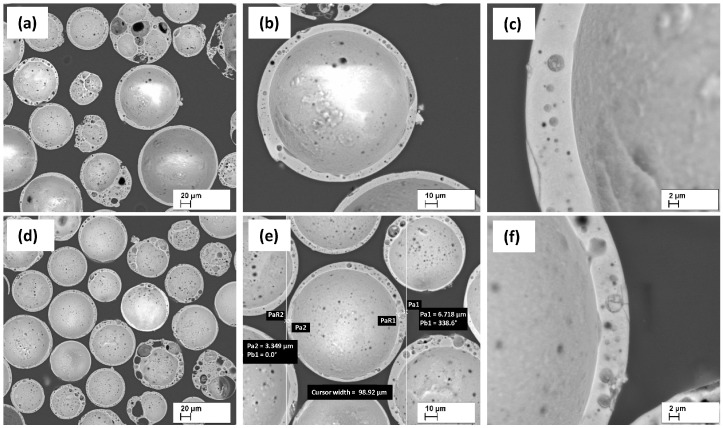
SEM micrographs of CS1 (**a**–**c**), and CS2 (**d**–**f**), at 200 (**a**,**b**), 500 (**b**,**e**), and 2000 (**c**,**f**) × times magnification, respectively. Adopted from [22] (CC BY Open Access, MDPI).

CS wall thickness and their structure are shown in Figure 1. CS have mainly spherical shapes and the CS wall thickness varies from 4 to 10 μm on average. As can be seen, the walls have closely spaced pores, with average pore sizes ranging from 0.5 to 5.0 μm. 

### 2.2. SPS Sintering

The samples were prepared for sintering in the SPS machine by lining a graphite mould with 0.35 mm graphite paper, inserting the bottom die and lining its surface in contact with the material to be sintered with 0.35 mm graphite paper, pouring in the needed amount of the selected CS, compacting the unsintered material by gently shaking the die, placing a 0.35 mm thick circular graphite paper on top of the sample to be sintered and inserting the upper graphite die into the mould. The outside of the mould was covered with a 4.5 mm thick, carbon fibre thermal insulating material and secured in place with a carbon fibre cord. The prepared mould was placed in the vacuum chamber of the SPS machine with three cylindrical graphite discs of an appropriate size on each side between the dies of the mould and the electrodes of the SPS machine, with 0.35 mm thick graphite paper between the largest of these discs and the electrodes of the machine. The mould was placed in a position where the axis of the pyrometer used to measure the temperature coincided with the temperature observation bore, so that the measured temperature was as close to the actual temperature of the sintered material as possible.

After inserting the mould into the SPS machine, the distance between the electrodes was reduced until the top electrode made contact with the top graphite disk and the shrinkage meter was zeroed. The die was subjected to the machine’s minimum clamping force, and the sample was held at this pressure for 10 min until the shrinkage due to compression of the sample stopped. The shrinkage gauge was then zeroed, the atmospheric pressure in the chamber was reduced to around 6 Pa, and the shrinkage was held for 20 min under these conditions until the vacuum-related shrinkage stopped. For SPS sintering, the temperature rise programmes were set to the same parameters between each batch of samples in all sintering processes: temperatures were raised at approximately 100 °C·min^−1^. To reduce the potential temperature rise above the sintering temperature and the resulting sample heterogeneity, the last 20 °C to sintering temperature were raised at 20 °C·min^−1^ to reduce the sample temperature rising above the expected sintering temperature, at which point the sample was held for 2 min. After the sintering process, cooling was carried out under vacuum for 20 min until the temperature of the sample reached approximately 300 °C. At that time, the vacuum chamber was opened to allow faster cooling. Ten minutes after opening the vacuum chamber, the sample was removed from the SPS machine and the graphite mould. A similar process was carried out for a series of samples with other sizes of CS and in other mould sizes, adjusting the size of the dies and the amount of material to be sintered accordingly.

### 2.3. Determination of the Quantity of Collapsed CS

The collapse of the CS associated with the compression and vacuuming steps of the sample in the SPS machine was determined by running experiments that were stopped after the compression or vacuuming step, respectively. The material in the mould was weighed using an VIC-612 balance (Acculab, Sartorius group, Goettingen, Germany). It was poured into a container and covered with deionised water, ensuring a water layer at least 5 cm thick. The mixture of material and water was mixed and allowed to settle for 1 h, followed by removing floating particles from the surface of the water using a paper towel to drag the particles over the edge of the container. Most of the water was decanted, and the sunken material was transferred to a smaller container, dried and weighed to determine the mass of the collapsed CS. 

The apparent density of the sintered samples was determined according to Formula (1) using an Acculab VIC-612 balance to determine the weight of the sample:

(1) $$\rho_{apparent}=\frac{4m}{\pi d^{2}h}$$

where: *d*—diameter of the sintered sample, mm; *h*—height of the sintered sample, mm; *m*—mass of the sintered sample, g.

To determine the open porosity of the resulting materials, they were sanded to remove the graphite paper adhered during the sintering process. The samples were then dried at 70 °C for four hours, after which their dry weight was determined using an analytical balance. The samples were boiled in distilled water for four hours and left for 15 h to allow the open pores to become saturated with water. The samples were weighed in the water-immersed state by using the above-mentioned balance equipped with an “AD-1653” density determination kit, after which their surface was dried with a piece of water-saturated cloth to remove water droplets from their surface before the saturated weight of the samples was determined. The open porosity, closed porosity, as well as the pore volumes, were then calculated using Formulae (2)–(6).

(2) $$V_{app}=M_{sat}-M_{sub}$$

(3) $$V_{op}=M_{sat}-M_{dry}$$

(4) $$V_{clo}=V_{app}-V_{op}-\frac{m_{dry}}{2.55}$$

(5) $$P_{op}=\frac{V_{op}}{V_{app}}$$

(6) $$P_{clo}=\frac{V_{clo}}{V_{app}}$$

where: *M_sat_*—mass of a water-saturated sample, g; *M_sub_*—mass of a sample submerged under water, g; *M_dry_*—mass of a dry sample, g; *V_app_*—apparent volume of the sample, ml; *V_op_*—volume of open pores, ml; *V_clo_*—volume of closed pores, ml; *P_op_*—open porosity; *P_clo_*—closed porosity, 2.55 is a pycnometric density of the CS wall material, in g·cm^−3^.

Due to the relative mechanical weakness of samples sintered at lower temperatures, in order to prepare them for optical microscopy, they were cast in epoxy resin. In this case, “Slip-LG 100” pouring epoxy system was used, obtained from Prestol Compozits, Riga, Latvia, securing the sample to a mould, pouring epoxy-hardener mixture over it and vacuuming it all down to 500 Pa pressure three times to extract air bubbles from the sample and fully saturate it with resin. After hardening, the samples were then sanded and polished to prepare the surface for optical microscopy.

The compressive strength of the sintered specimens was determined using ToniNORM model 2020 compressive strength tester, (Toni Technik GmbH, Berlin, Germany). In the test, the hydraulic cylinder of the machine applies a progressively increasing force to the specimen, increasing the compression pressure at a rate of 0.04 MPa·s^−1^ until the recorded compression force momentarily decreases by 1.2% due to specimen collapse. 

All samples for XRD analysis whose particle size is under 100 µm were ground into powder by using agate mortar and pestle, so that accurate powder XDR analysis could be performed. The unsintered CS sample used to determine the magnetic phase present in them was additionally concentrated from the samples used to determine the magnetic CS fraction in the unsintered material; this was performed by grinding them, and then using a neodymium magnet and distilled water to wash away anything that was not attracted to the magnetic field.

XRD image acquisition and qualitative analysis of the unsintered material and sintered samples was performed using D8 Advance (Bruker, Billerica, MA, USA), with a copper anode X-ray tube (40 kV voltage and 40 mA current). Diffraction images were taken between 10° and 80° 2θ, as a locked-coupled scan at a scan rate of 0.15° 2θ min^−1^ and a data registration step of 0.02° 2θ. The crystalline phases observed in the diffraction patterns were analysed using Diffrac.EVA 6.1 data processing software and the electronic COD (Crystallography Open Database) database. Diffraction image preparation was performed using Diffrac.EVA software to perform Fourier smoothing for the scans and to position them, and used GIMP version 2.10.34 to thicken the lines and add phase markers and scan numbers.

The magnetic fraction of the sinterable material was separated and determined for CS 63–150 µm and CS 150–250 µm particles. A 30 g thin layer of the test material was spread on a sheet of paper, and a cylindrical neodymium magnet was repeatedly moved close to its surface, the particles adhering to its surface were periodically removed and transferred to a separate container. The separated fractions were then weighed, and the fraction of the magnetic fraction was calculated according to the Formula (7):

(7) $$W_{mag}=\frac{m_{mag}}{m_{mag}+m_{nonmag}}$$

where: *m_mag_*—a mass of the magnetic fraction of the CS, g; *m_nonmag_*—a mass of the non-magnetic fraction of CS, g.

## 3. Results and Discussion

### 3.1. Particle Destruction Prior to the SPS Process

From the shrinkage results recorded, it can be seen that the samples also show significant shrinkage before the start of the process, i.e., at the time of application of the compression force and vacuum generation in the SPS chamber, approximately 9.7% and 24.5% of the initial height of the sample in the die, respectively, in the case of a 20 mm mould. This shrinkage before the start of sintering can be explained by the collapse of the CS as a result of the applied force, as confirmed by the flotation of the non-sintered CS in water after force and vacuum application in the SPS machine, which showed a significant proportion of collapsed CS, 21% after force application and a further 20% after vacuum application, of the initial mass of material. When the process was carried out in a 50 mm die which had a lower pressure exerted on the CS, the shrinkage of the sample during pressing was only 0.88 mm and no further shrinkage occurred during vacuuming, reducing the fraction of broken CS to 3%. The main reason for this was most likely the reduction in the pressure exerted on the samples from 2.2 MPa in the 20 mm mould to 0.9 MPa in the 50 mm one. Testing of 150–250 µm CS in the 20 mm mould, however, revealed that, similar to the 63–150 µm particles, 23% of the CS were destroyed after the force application step, while an additional 37% of the CS by mass were crushed after the chamber vacuum—significantly more than for the smaller particle fraction.

### 3.2. Sample Shrinkage during the SPS Process

**Figure 2:**
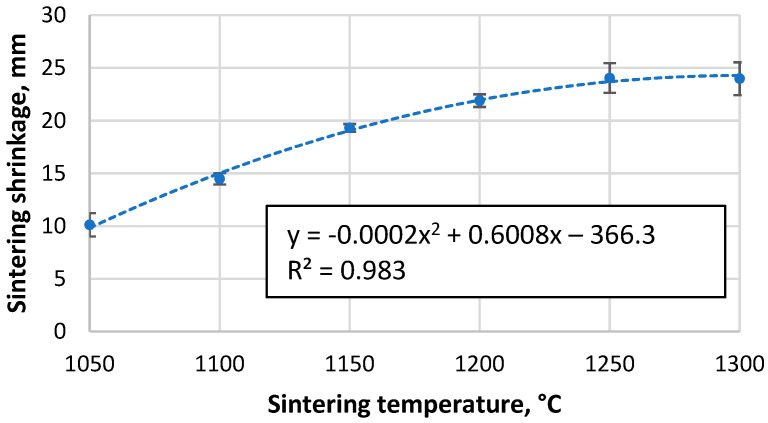
Shrinkage of CS 63–150 µm samples sintered in a 20 mm mould during the SPS process as a function of sintering temperature.

**Figure 3:**
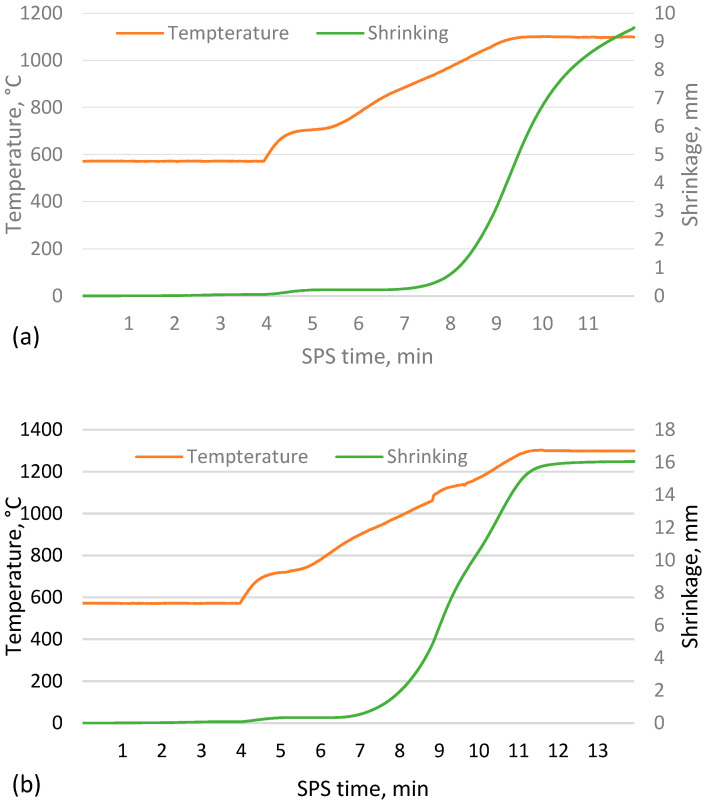
Sintering process data for CS 63–150 µm samples sintered in a 20 mm mould for 2 min at 1050 °C (**a**) and 1300 °C (**b**).

As shown in Figure 2, as the sintering temperature increases, the height of the sample decreases due to more compaction and softening of the particles, which reduces the open and closed porosity, allowing more shrinkage of the material. The data indicate a tendency for the shrinkage to reach a given value at higher temperatures, when nearly fully compacted samples are created and a rapid drop to zero, implying that sintering is worse at lower temperatures and that the apparent density of the resulting material will approach that of the bulk density of unsintered CS. However, this is not the case, as the CS did not form a cohesive material when attempting to sinter at 1000 °C, which is supported by the sintering data in Figure 3a,b, where it is observed that the shrinkage of the samples starts at approximately 900 °C. However, as the temperature rises at this speed, there is a significant difference between the measured and actual sample temperature due to the thermal inertia of the mould.

Analysis of the sintering process data shown in Figure 3a,b shows that at the lower sintering temperatures, the process is stopped before the shrinkage of the sample is complete, i.e., the particles are fully sintered. In comparison, at higher temperatures, the shrinkage stops briefly after the sintering temperature is reached. This can be explained by the softening of the CS caused by the increased sintering temperature, which allows the sample to reach equilibrium more quickly at the same compression force on the sample. 

**Figure 4:**
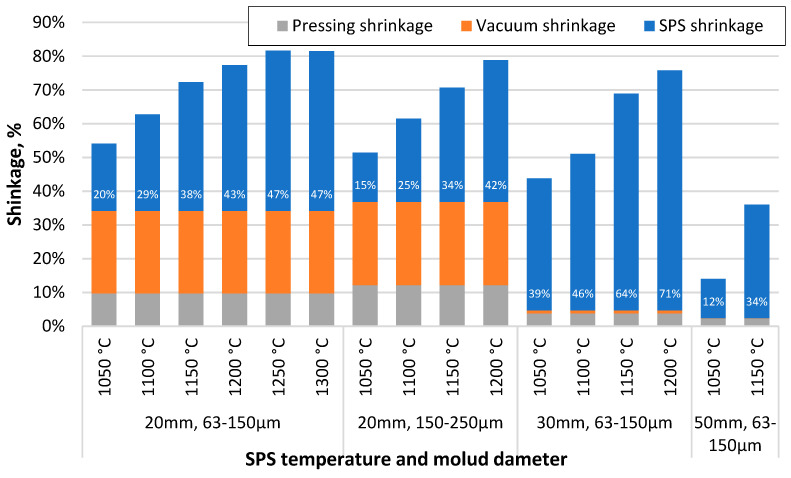
Shrinkage of specimens after squeezing, vacuum and SPS process steps, depending on sintering temperature, material used and die diameter.

Comparing the shrinkage of the samples at different stages of the sample creation process, some peculiarities can be observed. Figure 4 shows that the samples formed from the 150–250 µm CS fraction have less shrinkage during the SPS process at lower sintering temperatures, but this difference decreases with increasing sintering temperature. The higher wall thickness of the particles can potentially explain this difference. In contrast, as the temperature increases and the particles become soft under its influence, the effect of the thicker walls in increasing their mechanical strength becomes negligible. The specimens sintered in the 30 mm moulds have reduced shrinkage during pressing and vacuum due to the lower pressure on the surface of these specimens due to their larger diameter. However, their shrinkage during sintering is significantly higher, probably due to the breakage or deformation of previously unbroken particles at elevated temperatures, in contrast to the samples sintered in the 50 mm moulds, where the pressure is even lower.

### 3.3. The Apparent Density of the Resulting Materials

**Figure 5:**
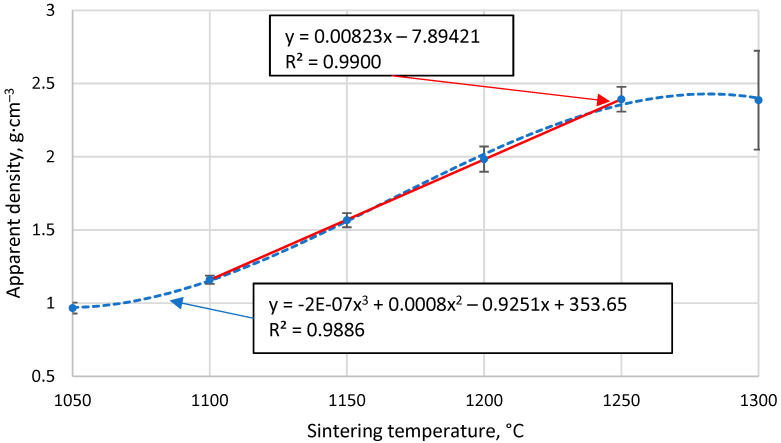
The apparent density of CS 63–150 µm samples sintered in a 20 mm mould as a function of sintering temperature.

Figure 5 shows that the apparent density of sintered cenosphere samples increases with increasing sintering temperature. However, this relationship is not linear over the entire data region, as 1050 °C is insufficient to allow complete sintering between the particles, while at 1300 °C, the particles become soft and are compressed. This means that if the temperature range over which sintering occurred were extended, a tendency towards the density of bulk CS would be observed at lower temperatures, while a tendency towards the proper density of the material forming the CS at higher sintering temperatures. Because of these facts, a non-linear regression analysis using a third-order polynomial will not be able to give an appropriate function for predicting the data, even if the R^2^ value of the resulting function is 0.989.

However, in the temperature range 1100 °C to 1250 °C, there is a marked linear increase in density with sintering temperature, as shown in Figure 5, giving R^2^ = 0.99 and a regression line function ρ = 0.0082x − 7.89 for the linear regression analysis. This means that over this temperature range and under these sintering conditions, it is possible to reliably predict the apparent density of the resulting materials by knowing their sintering temperature.

An important observation is that when looking at the whole set of samples produced and the individual batches, there is no linear relationship between the shrinkage of the samples and their apparent density, as would be expected from the formula for calculating the apparent density. Therefore, instead of estimating this relationship, it is possible to do so for the relationship between the measured apparent density and the predicted apparent density based on the observed sample shrinkage.

**Figure 6:**
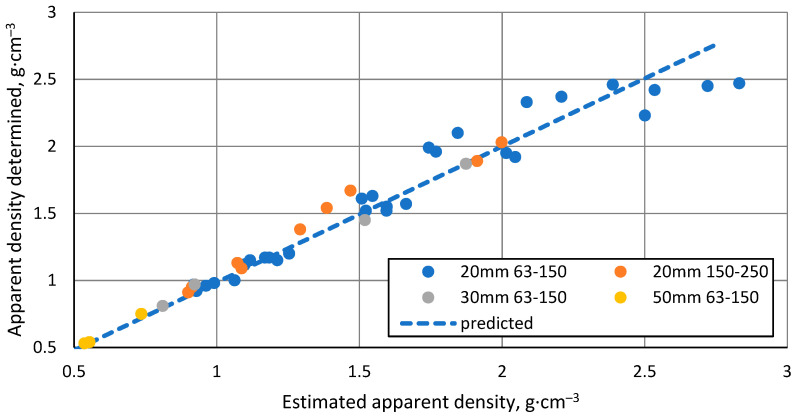
Relationship between predicted and observed apparent density of samples.

As can be seen from Figure 6, the relationship between the predicted and the determined apparent densities is approximately linear, which means that the apparent density of a sintered sample can be predicted by knowing the shrinkage of the sample to be sintered at all stages of the process, its mass and height before starting the process and the internal diameter of the mould. It also indicates that the material does not lose significant mass during sintering. The increased scatter in the data at the higher predicted apparent densities are due to the increased errors in determining the apparent densities caused by the reduced sample volume and the slight material bleed between the mould and its plunger at the higher sintering temperatures.

### 3.4. Porosity of the Resulting Materials

**Figure 7:**
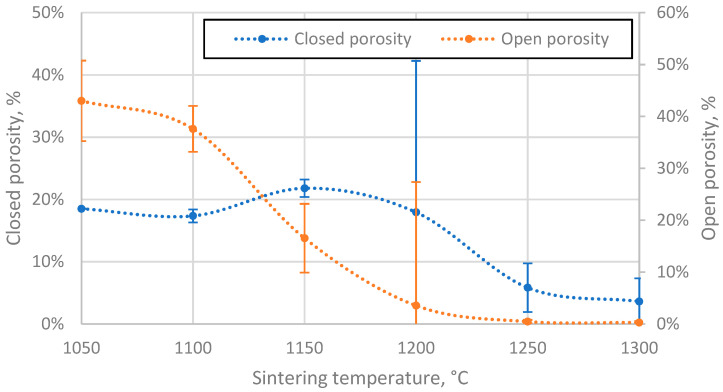
Open and closed porosity of CS 63–150 µm samples sintered in 20 mm mould as a function of sintering temperature.

The open porosity of the samples decreases with increasing sintering temperature, resulting in samples with open porosity ranging from 0.28% to 43% (Figure 7). However, this relationship is not linear, with the steepest drop in value occurring in the region between 1100 °C and 1150 °C, most likely because the CS particles in this region become soft enough to be slightly deformed and, therefore, more efficiently packed, thus reducing the inter-cenospheric space and at the same time closing some of the open pores. The decreasing speed of open porosity reduction observed with increasing sintering temperature can be explained by the increasing difficulty in packing the particles more densely.

**Figure 8:**
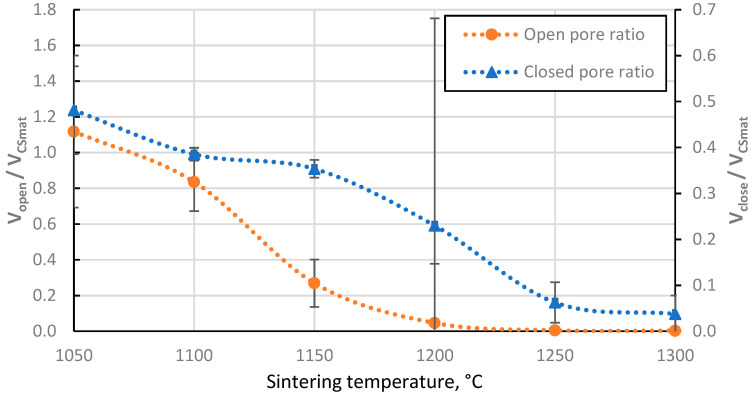
The ratio of closed and open pore volumes of sintered CS 63–150 µm samples to the volume of CS material volume (aluminosilicate only, excluding voids) as a function of sintering temperature.

The closed porosity of the sintered materials peaks in the sintering temperature region between 1100 °C and 1200 °C, as shown in Figure 7. This can be explained by the significant difference in the rate of closed and open pore volume reduction in this temperature region, as shown in Figure 8, where it can be observed that the closed pore volume reduction rate is highest in the temperature region between 1150 °C and 1250 °C, as opposed to 1100 °C and 1150 °C for open pores. This means it is possible to produce CS ceramics with a specific ratio of open to closed pore volumes if required (Figure 8).

**Figure 9:**
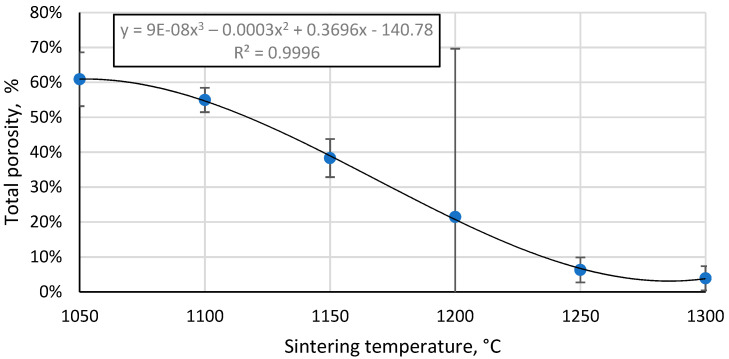
Total porosity of CS 63–150 µm samples sintered in a 20 mm die as a function of sintering temperature.

The total porosity of the sintered samples decreases with increasing sintering temperature (Figure 9), and there is a strongly linear decrease between 1100 °C and 1250 °C, as would be expected with the linear increase in the apparent density of these samples in this temperature range, since total porosity and apparent density are correlated values. Moreover, this indicates that no gross errors were obtained during the porosity determination.

### 3.5. Morphology of the Resulting Materials

**Figure 10:**
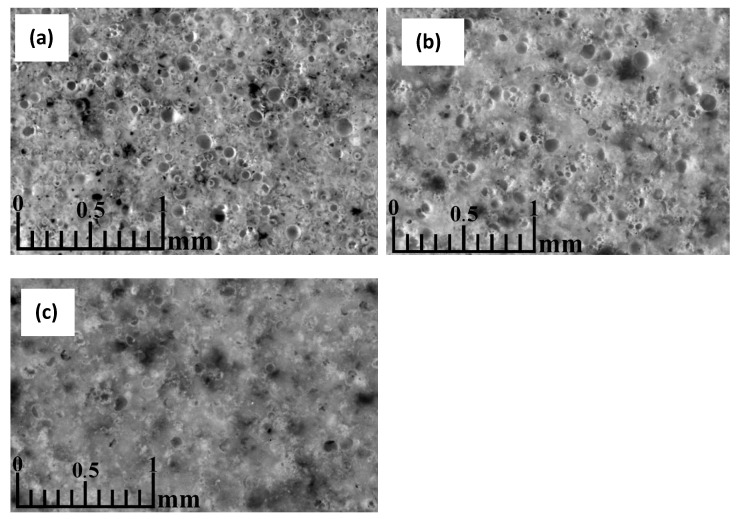
Optical microscopy image of CS 63–150 µm samples sintered in a 20 mm mould at 1050 °C (**a**), 1200 °C (**b**), 1250 °C (**c**), perpendicular to the die axis.

An optical microscopy image of a CS sample sintered in a 20 mm mould at 1050 °C (Figure 10a) shows that most of the CS at this temperature were not completely shattered or deformed during the sintering process, indicating that the softening point of the CS was not reached. Some of the CS have fusion bridges, holding the particles together. However, the amount of these bridges is relatively tiny when assessed visually, which accounts for the low mechanical strength of the samples sintered at 1050 °C compared to those sintered at higher temperatures. At this magnification, there is no marked difference in the sample structure between the edge and centre regions, indicating that no marked temperature or compressive force gradient across the sample volume was generated during the sintering of this sample, similar to the sample that was sintered in a 20 mm mould at 1200 °C (Figure 10b). Some undestroyed CS show translucent bubbles, the most likely origin of which is the partial penetration of epoxy into the CS during the impregnation of the sample, indicating holes in these particles. These holes could have been caused by the CS being too small to fill with water during flotation, cracks in their walls during pressing or vacuuming, or internal stresses as the sample cooled after sintering.

The sample sintered at 1200 °C (Figure 10b) shows deformation of the CS, indicating soft melting of these particles. This is likely to be the reason for the formation of the compacted regions: some of the hollow particles became soft enough during sintering that the applied compressive force could flatten them to form dense regions of material. This theory is also supported by the high shrinkage of the sample during sintering and its increase with increasing sintering temperature. It can be observed from the images of the sample that the magnetic CS, shown as darker particles in the images, have melted during the sintering of the sample sintered at 1200 °C, in contrast to the sample at 1050 °C, forming regions of higher magnetite content in the fused regions of the material. In addition to the undestroyed CS, a relatively large amount of plenospheres is also observed. The outer walls of these particles have melted with the bulk of the material. In contrast, the particles within them have mostly retained their shape and are distinguishable from the bulk material.

The sample sintered at 1250 °C (Figure 10c) shows a meagre amount of closed pores, mainly formed by deformed particles within the cenosphere and plenosphere, similar to the sample sintered at 1200 °C and much larger solid melt regions.

**Figure 11:**
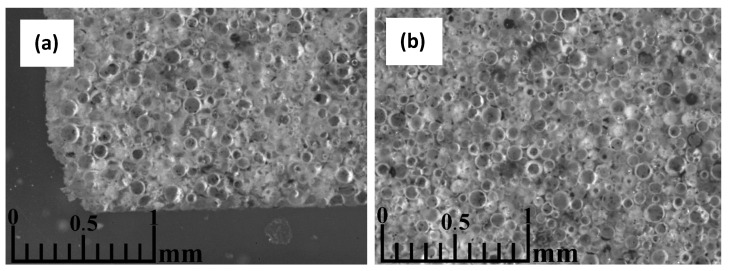
Optical microscopy images of a CS 63–150 µm sample sintered at 1050 °C in a 50 mm mould, parallel to the die axis, close to the material-mould boundary (**a**), and near the centre of the specimen (**b**).

Figure 11a shows that a denser layer of melt material, approximately 100 µm thick, has formed at the interface between the mould and the material to be sintered in the vicinity of the plungers. There are also better and worse-sintered regions throughout the volume of the material, which can be explained by the preferential formation of current flow channels during the sintering of the material. Although the unsintered material usually is not electrically conductive, from the current–voltage graphs of the process, there seems to be evidence of partial current flow through the CS during sintering at higher temperatures. As shown in Figure 11b, the sintering in the centre of the sample was significantly worse. It cannot be seen here, but the material’s colour there did not change from the slight red of the unsintered material to the greyish colour of the successfully sintered samples. This indicates a potential temperature non-uniformity in the sample during sintering, probably caused by the particles’ low electrical conductivity and low thermal conductivity, leading to the heating of the material mostly from the outside. This could probably be corrected by increasing the sample sintering time or decreasing the heating speed, thus allowing it to be fully heated. However, if the incomplete sintering of the particles in the centre of the sample was caused by an uneven distribution of force in the bulk material during the SPS process, the only solution would be to increase the squeezing force, which significantly increases the shrinkage and hence the apparent density of the material, or to reduce the h/d ratio of the sample several times, which would mean that the manufactured material would be limited in its thickness.

### 3.6. Compressive Strength of the Resulting Materials

**Figure 12:**
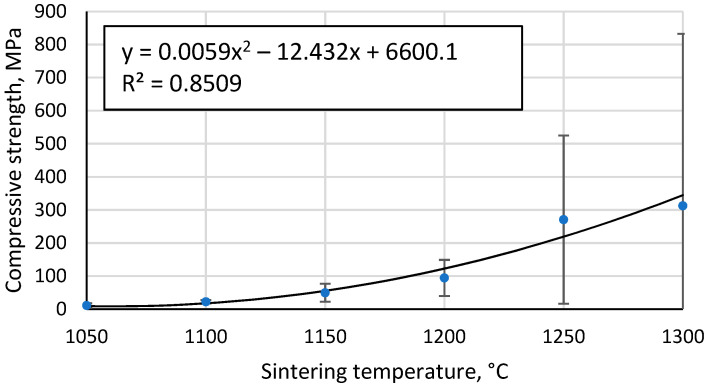
Compressive strength of sintered CS specimens as a function of sintering temperature.

All tested specimens are brittle, i.e., their collapse occurs spontaneously and is not preceded by the formation of large cracks in the specimens or deformation. However, small pieces of ceramic were, in some cases, detached from the samples and sintered at the highest temperatures before destruction due to insufficient homogeneity of the samples or the inhomogeneous contact surface between the sample and the testing apparatus. Figure 12 shows that the compressive strength of the samples increases with increasing sintering temperature, the most likely explanation being a combination of two effects. Firstly, higher binding between the CS particles, an effect that would be more pronounced at lower sintering temperatures where this binding between the particles is relatively weak, and secondly, the relationship between the density of the high-porosity materials and their mechanical strength.

**Figure 13:**
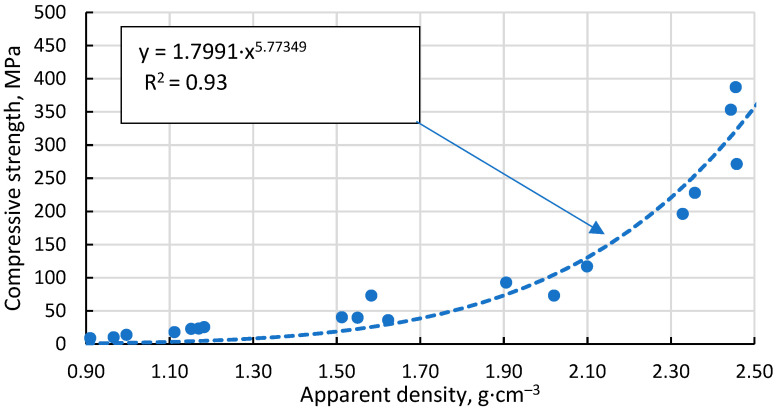
Dependence of compressive strength on apparent density of sintered specimens.

**Figure 14:**
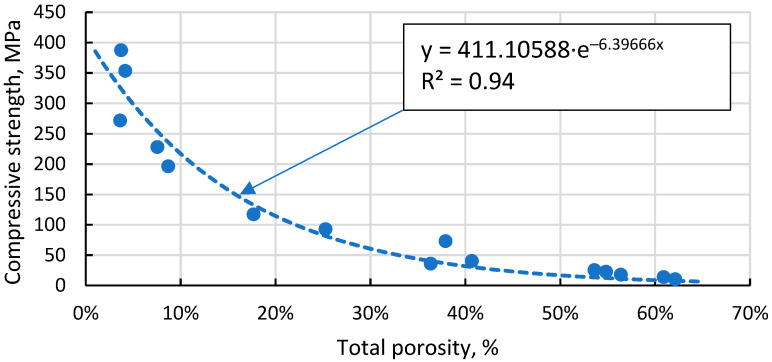
Dependence of compressive strength on total porosity of sintered samples.

The Gibson–Ashby model for predicting the properties of lattice type and porous materials [46], which is reducible to a power function, does not allow a conclusive correlation between the compressive strength results obtained for the specimens, giving R^2^ = 0.93 (Figure 13). An additional problem is the lack of reliable data on which to base the compressive strength and density of a fully compacted, sintered cenospheric sample. This means that the model cannot be used in this case. The model suggested by Rice [47] gives a visually and mathematically better agreement with R^2^ = 0.94 (Figure 14). In addition, this model also gives a reliable value for the compressive strength of a dense material, σ_s_ = 411.1 MPa since, as mentioned above, these values are not known.

### 3.7. Composition of the Crystalline Phases of the Starting Materials and Samples

**Figure 15:**
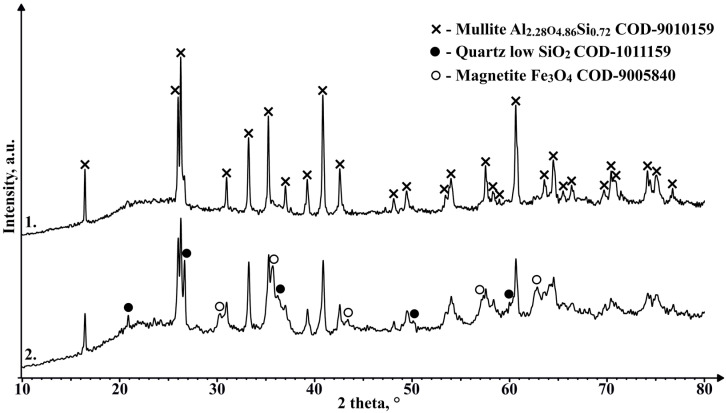
XRD results of magnetic CS 150–250 µm fraction (1), and magnetic particle concentrate from that same fraction (2).

Figure 15 shows that the crystalline phase that makes some of the CS ferromagnetic is magnetite. However, the concentration of it even in magnetic CS is extremely low, hence the need for the additional concentrating of magnetic particles.

**Figure 16:**
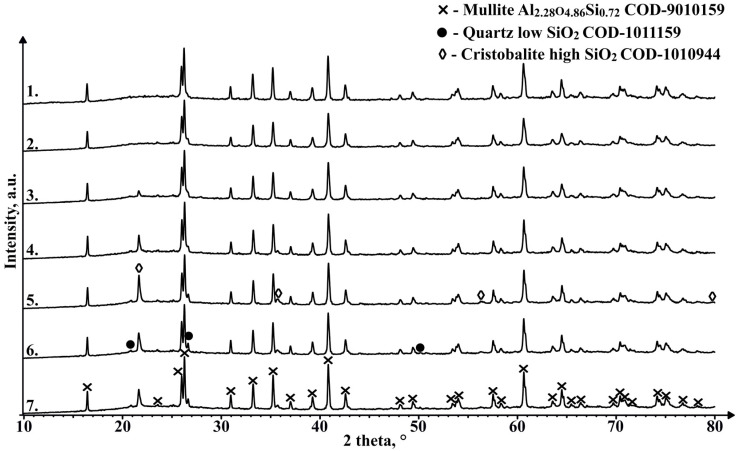
XRD results of unsintered CS2 63–150 µm (1), and samples sintered in a 20 mm mould for 2 min at 1050 °C (2), 1100 °C (3), 1150 °C (4), 1200 °C (5), 1250 °C (6) and 1300 °C (7).

As can be seen in Figure 16, there are no marked differences in phase composition between the unsintered material and the 1050 °C sintered sample, with the main components being mullite and an X-ray amorphous phase in the region between 18° and 27° 2θ, most likely caused by the amorphous part of the CS, which is mainly composed of SiO_2_. The samples sintered at 1100 °C and higher temperatures show an additional phase with a diffraction pattern consistent with cristobalite. The amount of this phase between samples that contain it differs and does not seem to be corelated with the sintering temperature, which could be caused by the fact, that the XRD analysis was not performed on an average sample of the sintered pieces. The appearance of this phase in the sample could be due to the partial melting and subsequent crystallisation of the amorphous regions of the CS due to the relatively slow cooling compared to that during the formation of the CS. Despite confirming the presence of a magnetic phase in some of the sinterable particles based on their interaction with the external magnetic field, this crystalline phase is not observed in the XRD results for an average unsintered CS sample, which can be explained by relatively low content of these phases. Similarly, a very weak interaction of the sintered samples with the external magnetic field is also observed, so some of the magnetic phase found in the material to be sintered has most likely been retained during the SPS process, or some other magnetic phase has been generated.

### 3.8. Comparison of Material Properties with Literature Data

Comparison of the samples obtained with those from other studies is difficult, as very few studies have obtained sintered CS with low levels of other added materials, nor have studies sintered CS using the SPS method. The closest case is copper-coated CS using vibration-assisted sputter coating, and titanium and titanium nitride-coated CS and sintered by SPS [39]. Comparing the apparent density–compressive strength relationship of the copper-coated CS with the samples obtained in this work, the strength of the uncoated CS is slightly lower, the difference increasing with the increasing apparent density of the material. On the other hand, the titanium and titanium nitride-coated CS simultaneously have a lower apparent density and a higher compressive strength over the comparable temperature range, which is probably due to the higher breakage of the CS before sintering starts in the case of the uncoated CS. 

In Table 3, presented data of lightweight composite by type a syntactic foam with CS is shown. A significant part of funded works describes materials (Nr.1–10 in Table 3) with an Al or Mg matrix. Data close to our study by criteria density are materials with densities from 0.89 to 1.55 g·cm^−1^, but its maximal working temperature, obviously, is limited by the Al or Mg alloy melting temperature (600–660 °C). Material Nr. 10 on the Ti matrix definitely has higher working temperature, potentially up to 1500 °C (Ti melting point is 1668 °C), but Ti intensively oxidised in air atmosphere above the 900 °C [48]. Material Nr. 23 (SPS at 1150 °C), studied in this work, is comparable by mechanical properties with Nr. 9 (Table 3) on the Mg matrix. However, it differs by the working temperature (limited to 660 °C). Materials on the polymer matrix (Nr. 20–22 Table 3) obviously has a significantly lower working temperature, which is limited by polymer matrix softening at 200 °C. Material examples Nr. 11–19 are quite close to the studied materials. However, all FSs on a clay matrix has a temperature limit up to 800–900 °C, due to clay softening. Most close to this present study is material Nr. 17. Proposed CS-SPS sintered material has an advantage in comparison to this material: a much quicker process—SPS took 2 min of sintering and 20 min in total for heat up and cool down; mullite powder is not necessary; and a higher working temperature.

## 4. Conclusions

Using the SPS method for consolidation of the CS, lightweight porous ceramics were obtained, with predominant closed porosity, which could be classified by the structure as syntactic foam. In sharp contrast with classical syntactic foam, the studied material does not contain any matrix material. For such material, it has been proposed to use the term, “matrix-less syntactic foam”. The obtained material at SPS temperature from 1050 to 1300 °C is characterised by porosity in a range from 0.5 to 2.8 g·cm^−3^. Varying SPS temperature is possible to obtain material with a tailored density.

Compression strength has a strong corelation with material apparent density, and depends on the sintering temperature: increasing sample sintering temperature increases the apparent density of all sample series—CS 63–150 µm in a 20 mm mould from 0.97 g·cm^−3^ to 2.3 g·cm^−3^ at 1050–1300 °C temperature; in a 30 mm mould, 0.81–1.87 g·cm^−3^ at 1050–1200 °C temperature; in a 50 mm mould, 0.54–0.75 g·cm^−3^ at 1050–1150 °C temperature; while CS 150–250 µm in a 20 mm mould is 0.93–1.96 g·cm^−3^ at 1050–1200 °C temperatures. Total porosity decreases from 61.5% to 3.9%, increasing the sintering temperature from 1050 °C to 1250 °C, while open starts to decrease at lower temperatures, closed porosity is the highest in samples sintered at 1150 °C. Increasing the sintering temperature from 1050 °C to 1300 °C, the compressive strength of CS 63–150 samples obtained in the 20 mm mould increases from 11 MPa to 312 MPa. These results correlate with the Rice model, which describes exponential compressive strength dependence on material porosity and completely dense material compressive strength.

The sample shrinkage before the beginning of the sintering process decreases with increasing mould diameter, which is caused by decreasing the breaking of CS, which can be explained by decreased pressure on the material. The sample shrinkage during the sintering process began at 900 °C, which shows that using the SPS method, the sintering of cenosphere particles starts between 900 °C and 1000 °C. Shrinkage of samples during sintering increases with increasing temperature and decreases with increasing mould diameter.

Material that is good enough in comparison with known materials by a set of properties (such as density, compression strength and thermal stability) was developed in this study. This material could be used for thermal insulation in extreme condition, where it is mandatory for all these properties to be present simultaneously, for example, in aerospace and defence fields.

## Figures and Tables

**Table 1:** Chemical composition of CS1, CS2 in wt. % [22].

Sample	SiO_2_	Al_2_O_3_	Fe_2_O_3_	CaO	MgO	Na_2_O	K_2_O	LOI * 400 °C, %	LOI * 1000 °C, %
CS1	56.5	36.9	1.4	2.4	1.2	1.1	0.5	0.5	0.1
CS2	53.8	40.7	1.0	1.4	0.6	0.5	0.4	0.6	0.4

* LOI—Loss on ignition.

**Table 2:** Bulk density of CS, the density of the material, and interparticle void fraction [22].

Material	Bulk Density, g·cm^−3^	Pycnometric Density g·cm^−3^	Voids, %
Raw CS1	0.415 ± 0.004	2.153 ± 0.001	40.0
Raw CS2	0.380 ± 0.002	2.272 ± 0.001	43.0

**Table 3:** Some composite materials with a density less than 2.0 g·cm^−3^, made using lightweight fillers.

Nr.	Used Materials	Density, g·cm^−3^	Melt./ Dec., °C	Compr. Strength, MPa	Porosity, %			Pore Size, µm	Sintering Method	Ref
					Total	Open	Closed			
	Metal matrix syntactic foam									
1	AlSi7Mg + light expanded clay agglomerate particles + Al_2_O_3_ or SiC	1.58–1.71	570	60–79				<2300	Low-pressure infiltration	[49]
2	AlSi7Mg + ceramic hollow spheres + Al_2_O_3_ or SiC	1.66–1.9	570	101–137						
3	Mg + Low density volcanic rock	0.89	650	10			49.5		Pressure infiltration	[50]
4	Mg + High density volcanic rock	1.68	650	40			32.5		Pressure infiltration	[50]
5	Mg AZ61 alloy + CS + carbamide granules	0.79–1.1	650	16–30				<1000	Microwave sintering	[51]
6	Al + G1.45 Globocer hollow spheres	1.8	660	43				1000	Low-pressure infiltration	[52]
7	Al + G3.83 Globocer hollow spheres	1.55	660	43				3500		
8	Mg + G1.45 Globocer hollow spheres	1.5	650	84				1000		
9	Mg + G3.83 Globocer hollow spheres	1.15	650	59				3500		
10	CS + Ti + NaCl + PVA	1.33–1.81	1500		50–63	50–60		<75	Argon atm. furnace	[53]
Ceramic matrix syntactic foam										
11	CS 60 vol.% + clay	0.94	900	7	66	28		50–100	Muffle furnace 1000 °C	[35]
12	CS 50 vol.% + clay	1,10	900	10	53	21				
13	CS 30 vol.% + clay	1,50	900	23	37	13				
14	Ceramic aerogel ZrB_2_	0.26–0.48		0.26–0.51		85–93		12–31	In-situ synthesis	[54]
15	Waste glass powder + incinerated sewage sludge ash	1.67–1.89	600	7–43		10–43		0.1–1.3	Muffle furnace	[55]
16	CS + ball clay + rice husk ash	0.57–0.67	900	5–8	50–56			<150	Muffle furnace	[56]
17	CS + mullite	1.18–1.86	900	2–187		56–18		<65	Box furnace	[57]
18	Cu coated CS	0.9–1.5	800	9–62	47–67			<200	SPS	[39]
19	CS + ferronichel slag	1.18–2.0		1.6–42	51–26			93–290	Microwave sintering	[58]
Polymer matrix syntactic foam										
20	CS + PVA	1.4–1.9	200	up to 100	>8.8			<20	Microwave sintering	[59]
21	CS + more PVA	0.54	200		39					
22	CS + PVA + PU	0.44	200	10	66			<250		
Matrix-less syntactic foam (this work) made in a 20 mm mould										
23	CS 63–150 µm	0.97	1100	10.95	61.5	43.00	18.51	50–120	SPS at 1050 °C	
23	CS 63–150 µm	1.16	1100	22.21	54.9	37.59	17.35		SPS at 1100 °C	
25	CS 63–150 µm	1.57	1100	49.57	38.3	16.53	21.80		SPS at 1150 °C	
26	CS 63–150 µm	1.98	1100	94.26	21.5	3.54	17.93		SPS at 1200 °C	

Melt./Dec.—Melting or decomposition temperature; PVA—Polyvinyl alcohol; PU—polyurethane.

## Data Availability

Data are contained within the article.
